# Supplementary material for: The interactome of intact mitochondria by cross-linking mass spectrometry provides evidence for coexisting respiratory supercomplexes
Source: Mol Cell Proteomics. 2017 Dec 8;17(2):216–32. doi: 10.1074/mcp.RA117.000470 (PMC5795388; doi:10.1074/mcp.RA117.000470)
Supplement: Supplemental Data [file supp_RA117.000470_133922_0_supp_23978_fzffwf.zip › spectra_annotation/mito_DR_spectra_annotation/144-1-1-1-1-1.pdf]

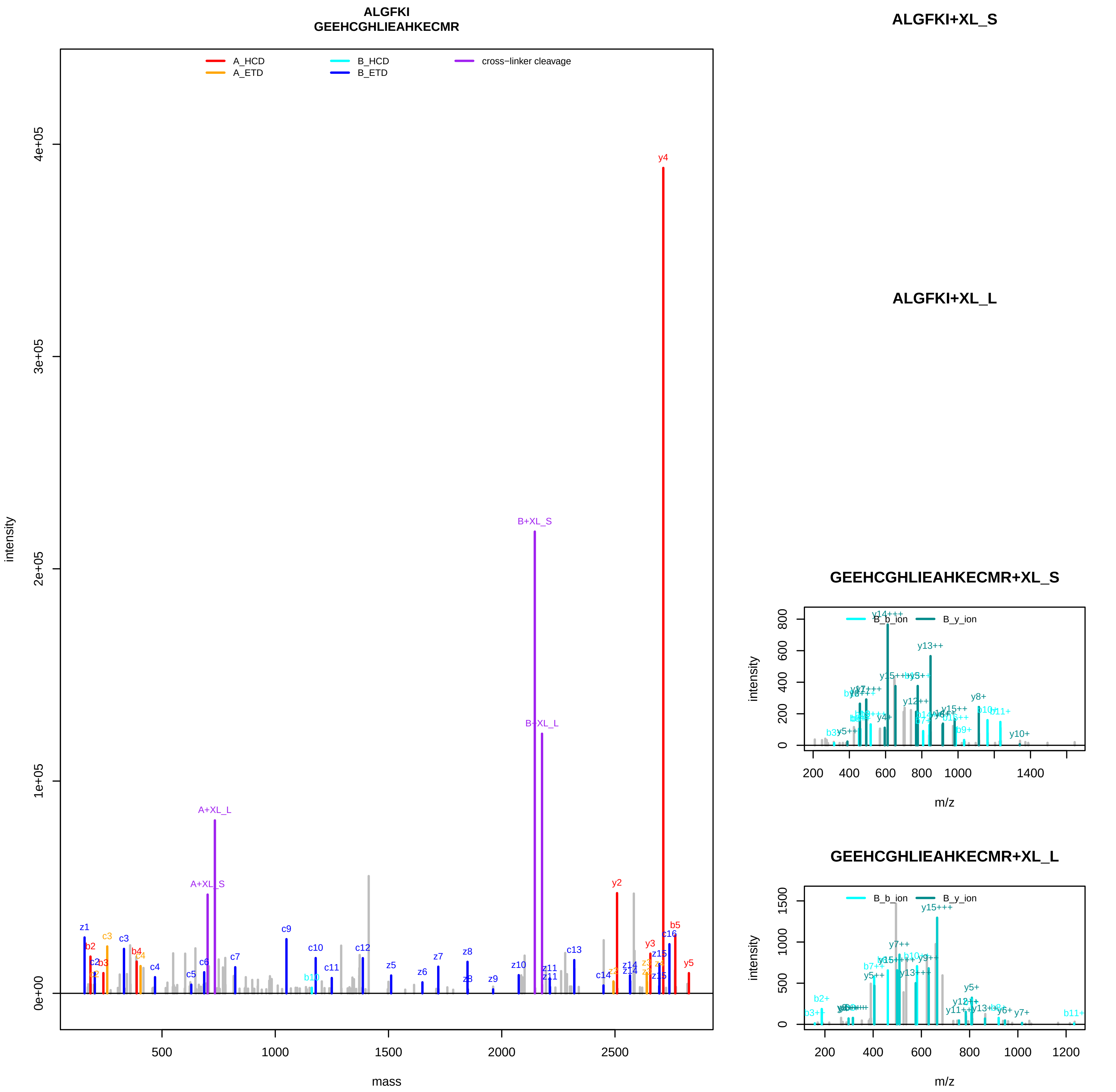

ALGFKI
GEEHCGHLIEAHKECMR
A_HCD
A_ETD
B_HCD
B_ETD
cross−linker cleavage
intensity
mass
0e+00
1e+05
2e+05
3e+05
4e+05
500
1000
1500
2000
2500
y4
B+XL_S
B+XL_L
A+XL_L
A+XL_S
y2
b5
y5
y3
c16
z15
c13
c14
z14
z11
z10
z9
z8
z7
z6
z5
c12
c11
c10
b10
c9
c7
c6
c5
c4
b4
c3
b3
b2
z1
ALGFKI+XL_S
ALGFKI+XL_L
GEEHCGHLIEAHKECMR+XL_S
B_b_ion
B_y_ion
intensity
m/z
y14+++
y13++
y12++
y8+
y10+
y15+++
y5++
y4+
b9+
b10+
b11+
b3+
GEEHCGHLIEAHKECMR+XL_L
y15+++
y7++
y5++
b7++
y13+++
y5+
y12++
y11++
y6+
y7+
b2+
b3++
b11+
0
500
1000
1500
200
400
600
800
1000
1200
1400
